# Mycobacterium tuberculosis infection in women with unexplained infertility 

**Published:** 2015-12

**Authors:** Maryam Eftekhar, Soheila Pourmasumi, Parvin Sabeti, Abbas Aflatoonian, Mohammad Hasan Sheikhha

**Affiliations:** *Research and Clinical Center for Infertility, Shahid Sadoughi University of Medical* *Sciences, Yazd, Iran.*

**Keywords:** *Genital mycobacterium tuberculosis*, *Polymerase chain reaction*, *Unexplained infertility*

## Abstract

**Background:**

Genital tuberculosis (GTB) is an important cause of female infertility, especially in developing countries. The positive results of polymerase chain reaction (PCR) in endometrial GTB in the absence of tubal damage raise the possibility of the detection of sub-clinical or latent disease, with doubtful benefits of treatment.

**Objective:**

To evaluate the mycobacterium tuberculosis infection in endometrial biopsy samples collected from unexplained infertile women attending Yazd Research and Clinical Center for Infertility by using PCR techniques.

**Materials and Methods:**

In this cross sectional study, 144 infertile women with unexplained infertility aged 20-35 years old and normal Histro-saplango graphy findings were enrolled. Endometrial biopsy samples from each participant were tested for mycobacterium tuberculosis detecting by PCR. In 93 patients, peritoneal fluid was also taken for culture and PCR.

**Results:**

The PCR results of endometrial specimens were negative in all cases, demonstrating that there was no GTB infection among our patients.

**Conclusion:**

Our results showed that GTB could not be considered as a major problem in women with unexplained infertility. Although, studies have indicated that PCR is a useful method in diagnosing early GTB disease in infertile women with no demonstrable evidence of tubal or endometrial involvement.

## Introduction

Infertility is typically defined as inability to achieve pregnancy after one year of unprotected intercourse (1, 2). The incidence of infertility shows marked variations in different countries ranging between 5 and 20% (3-7).

Previous investigations revealed that female infertility is caused by various factors including: ovulation problems, infections in the genital areas (8-10), tubal blockage (8, 11), uterine problems (8, 12), endometriosis (8, 12), pelvic infections (8, 13), endocrine disorders (8, 14, 15), and aggregated factors (8, 16, 17). However, there is another type of female infertility referred to as ‘Unexplained Infertility’, which in a case of infertility, medical examinations do not reveal any specific cause for infertility (16-19).

Genital tuberculosis (GTB) is one form of extrapulmonary mycobacterium tuberculosis (MTB) and is responsible for a considerable proportion of female infertility, especially in developing countries (20, 21). According to existing reports, 5–10% of infertile females all over the world have GTB; the incidence varies from less than 1% in the industrialized countries to nearly 13% in the developing countries. In most cases, GTB affects young women between the ages 20 and 40 years (22). In our literature review, it could not be found any accurate statistics of GTB among Iranian infertile patients. However, in a number of studies done in Pakistan, the frequency of GTB has been 2-10% (23-25). A study was done in Pakistan has shown that the infertility frequency in patients with GTB is 42.5% (26). It is similar to other countries such as India, where the frequency of GTB is reported as 3% in infertile women and 41% in tubal factor infertility (27).

Mani *et al* (2003) found that diagnosis of GTB by Ziehl-Neelsen stain could be achieved only in 2% of cases (28). While Biswas *et al* in suspected genital MTB cases detected the positive results in 4.8% of cases by culture, but none by Ziehl-Neelsen staining (29).

A study by Abebe *et al* reported that 4% of the clinically suspected patients of GTB were positive by Acid-Fast Bacilli staining, 12% by culture and 28% by histopathology, while polymerase chain reaction (PCR) gave the highest detection rate of 48% (30).

The pathogen spreads to the fallopian tubes (in 92-100% of cases), endometrium (in 50% of cases), ovaries (in 10-30% of cases), cervix (in 5% of cases), vagina, and vulva (in less than 1% of cases) leading to a variety of clinical presentations such as chronic lower abdominal or pelvic pain, vaginal bleeding, menstrual irregularity, general malaise, and infertility (22, 31, 32). When the female genital organs are affected by mycobacterium tuberculosis, it produces devastating effects by causing irreversible damage to the fallopian tube resulting in infertility (20, 22, 31, 32).

Therefore, GTB not only causes tubal dysfunction and obstruction, but also impairs implantation due to endometrial involvement and ovulatory failure from ovarian involvement (22, 32).

A risk of GTB should always be considered for young women presenting with unexplained infertility or repeated in vitro fertilization cycles failure. Clinical diagnosis of genital MTB is difficult, since asymptomatic latent cases predominate over symptomatic ones. Therefore, a high degree of suspicion and intensive investigations is important for diagnosis of GTB (7, 9).

Routine laboratory examinations, which are frequently used for GTB diagnosis are; chest X-ray, tuberculin test, Histro-Salpango Graphy (HSG) for beading, sacculation, sinus formation and a rigid “pipestem” pattern of the fallopian tubes, histological examination by acid-fast stain, and culture of biopsies obtained by laparoscopy or endometrial curettage (23-25).

HSG and laparoscopy remain the initial diagnostic procedures in the assessment of tubal and peritoneal factors leading to infertility (33). Absolute GTB diagnosis could not be made from characteristic features in HSG or laparoscopy. Laparoscopy is a valuable procedure only for obtaining tissue for histopathological examination, or for mycobacterial culture and PCR (33).

It will be important to utilize highly sensitive methods for GTB to diagnose the disease reliably in its early stages. In recent years, PCR has evolved as a useful and rapid technique for diagnosis of GTB (23-25). This technique has been adopted by many laboratories because of high sensitivity and specificity in comparison to other diagnostic methods. The aim of the present study was to evaluate MTB infection in endometrial biopsy samples collected from unexplained infertile women with no demonstrable evidence of tubal or endometrial involvement, attending our clinic. Therefore, based on these findings, this study was performed the test the MTB infection among infertile women by PCR. 

## Materials and methods

This study was conducted after obtaining ethical clearance from The Ethics Committee of Yazd Research and Clinical Center for Infertility. In this cross sectional study, the infertile women aged between 20 -35 years with normal HSG findings, referred to Yazd Research and Clinical Center for Infertility between January 2009 and March 2011 were enrolled. After signing an informed consent form, finally 144 infertile women were studied.

The subjects with a history of endocrine disorders, polycystic ovarian syndrome or any known cause of infertility were excluded. Also, women with prior history of tuberculosis, contact history of tuberculosis, and chest radiological findings were excluded, too. 

Hysteroscopy was done on the remaining subjects to look for status of fallopian tubes, presence of cornual or fimbrial block, tubal beading, presence of tubercles, tuboovarian masses, peritubal, periovarian adhesions, rigid tubes, fimbrial phimosis, hydrosalpinx, bowel, omental adhesions, intrauterine adhesions, small uterine cavity, and fibrosed ostia. 

Finally, 144 women who had normal HSG, and follicle-stimulating hormone level <10 IU/ml, were included in the study, and tested for MTB detecting in endometrial biopsy samples by PCR.

**Specimen collection**

Endometrial biopsy/curettage samples were conducted on the late follicular phase of menstruation under aseptic conditions. In 93 patients, at the time of laparoscopy, 20 ml of fluid aspirated from the pouch of Douglas, or after washing, collected into sterile tubes, and sent for culture and PCR study.

**DNA extraction**

DNA extraction from endometrial tissue samples (fetal tissues and placentas) was performed using a commercial kit (AccuPrep Genomic DNA Extraction Kit, Bioneer, S. Korea) following the manufacturer’s instructions. Briefly, 100μL of thawed homogenates of tissues were mixed with 600μL of Nuclei Lysis Solution and homogenized for 10 seconds. Samples were incubated at 65^°^C for 30 min, followed by the addition of 17.5μL proteinase K (20mg/ml) and incubation at 60^°^C for three hours, vortexing every 30 min. Three microliters of RNaseA (4mg mL-1) were added and the samples were mixed and incubated at 37^°^C for 30 minutes. After cooling, 200μL of the protein precipitation solution were added followed by vortexing and centrifugation at 13,000 g for 4 minutes. The supernatant was transferred into a new micro tube with 600μL of isopropanol, mixed, and centrifuged at 13,000 g for 3 minutes. The supernatant was discarded and the pellet was washed with 600μL of 70% ethanol, followed by a final centrifugation at 14,000 g for 3 min. Each pellet was dissolved in 100μL of the DNA rehydration solution by incubating at 65°C for one hour. DNA quality was assessed by spectrophotometry and PCR amplification of an internal control (β-Globulin gene). Samples did not yield a β-Globulin amplicon and had DNA concentration lower than 100ng/μL were excluded from further analysis.

**Polymerase Chain Reaction (PCR)**

DNA samples obtained from endometrial specimens were PCR tested for detection of Mycobacterium tuberculosis. PCR reactions were performed using a Cinagen diagnostic kit (Cinagen Company, Tehran, Iran) according to manufacturer’s instruction.

Bacterial DNA amplified in Eppendorf PCR machine (5860 Gradient Eppendorf Master Cycler, Germany). 

Final products of PCR were resolved by electrophoresis in a 1% agarose gel stained with ethidium bromide. Samples were determined to be PCR-positive when the 163bp MTB DNA fragments were presented on gel and were PCR- negative when this fragment was absent.

Positive controls included DNA from cultured organisms or infected tissues and negative controls in which DNA template was replaced by PCR-grade water were included in all reactions. The obtained PCR results were retrospectively correlated with the laparoscopic features of the individual patient.

**Statistical analysis**

The collected data were analyzed by statistical analysis using Statistical Package for the Social Sciences, version 16.0, SPSS Inc., Chicago, Illinois, USA. The data were expressed as mean ± SD, p value ≤0.05 was accepted as significance.

## Results

The mean (SD) age of all participants was 29.7 (4.14) years (range: 19 to 41). The demographic, clinical and endocrinological characteristics of participants are showed in tables I and II.

**Table I:** Demographic characteristics of the study samples (n=144)

**Variable**	**Category**	**Studied patients n (%)**	**p-value**
**Age (years)**	20–24	29 (20.2)	0.803
	25–29	63 (43.7)	
	30–34	52 (36.1)	
**Living place**	Urban	110 (76)	0.10
	Rural	34 (24)	
**Educational level**	Not educated	9 (6.3)	0.725
	Elementary	29 (20.2)	
	High School	67 (46.5)	
	College	39 (27)	
**Occupation**	Housewife	52 (36.1)	0.169
	Worker	28 (19.6)	
	Employees and teachers	64 (44.3)	

Chi-square and Student’s* t* test were used. P value ≤0.05 was accepted as significance.

**Table II.** Clinical and endocrinological characteristics of the study participants (n=144

**Variable**	**Means ± SD**
**Age (year)**	29.7 ± 4.14
**Infertility duration (year)**	4.93 ± 2.35
**Basal FSH (IU/ml)**	7.28 ± 0.6

All data are presented as mean ± standard deviation (SD).

FSH: follicle-stimulating hormone.

**Figure 1:**
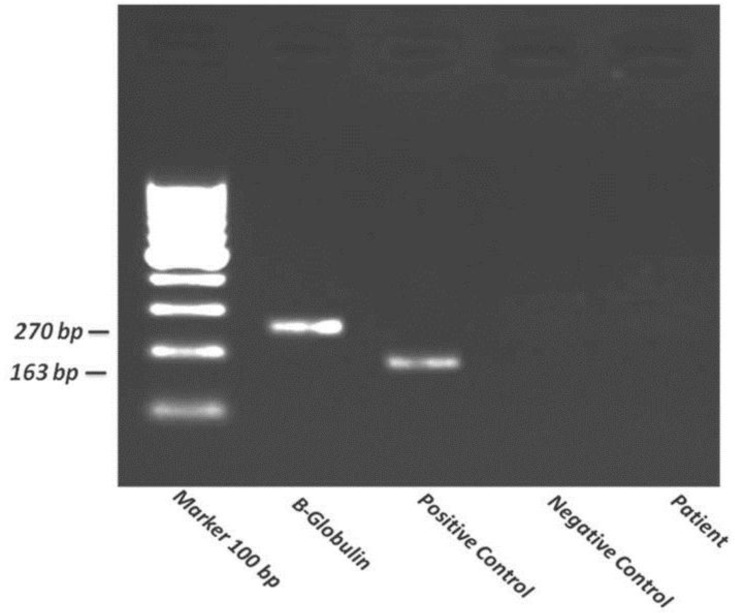
PCR product of amplified mycobacterium tuberculosis

It was not found any cases with MTB DNA amplification in endometrial specimens PCR results, which assumed as the gold standard method, demonstrating there was no GTB affection among our patients. PCR product of amplified Mycobacterium tuberculosis was run on 1% agarose gel. Positive control showed expected 163 bp fragment. There is no DNA amplification in patient and negative control. B-Globulin was used as internal control (270 bp) and marker of 100 bp size was run for control (Figure 1).

## Discussion

GTB is a well-recognized entity in the etiology of infertility, especially in low-income countries like India, Nepal, Bangladesh, Saudi Arabia, Nigeria, Sudan and Pakistan, where MTB is a prevalent disease (20, 22, 34, 35). The prevalence of GTB in women presenting with infertility varies from 1% in developed countries to 30% in developing countries (20, 34-36). According to the latest World health organization report from Iran in 2009, annually 9763 new cases of TB recorded, and about 28% all of this cases are extra pulmonary tuberculosis, GTB are also in this group (37).

However, in this study in a sample of Iranian unexplained infertile women, GTB was negative. The actual frequency of GTB is unknown despite different published data from various countries as it is often discovered incidentally or remains ‘undetected’ in symptomless patients (20, 22, 34). 

The primary focus of GTB is the fallopian tubes. The tubes are thickened and shown a rough external surface with adhesions. Caseous ulceration of the mucosa produces ragged contours and diverticular outpouchings of both isthmus and ampulla. The obstruction of the tube most frequently occurs in the region of transition between the isthmus and the ampulla. Hydrosalpinx is also a common finding in GTB (20, 22, 32), but it was not found any abnormalities such as cornual or fimbrial block, tubal beading, presence of tubercles, tuboovarian masses, peritubal and periovarian adhesions, rigid tubes, fimbrial phimosis, hydrosalpinx, bowel and omental adhesions, intrauterine adhesions, small uterine cavity, and fibrosed ostia in HSG of our patients. Hatami performed a 20-year retrospective study of Iranian genital tuberculosis and, reported that about 52 cases had genital tuberculosis among them 27 cases (52%) suffered from infertility. Also in about 70% of cases fallopian tubes were open (38). Tubal MTB spreads to the endometrium in one half of the cases. Distal tubal disease appears secondary to peritubal adhesions. Endometrial MTB has been reported to have a nonspecific appearance on HSG, commonly characterized by synechiae, a distorted uterine contour, and venous and lymphatic intravasation, but they were not found in our study. Early detection of GTB is crucial because once the infection has damaged the tubes; restoring tubal patency is very difficult (20, 22, 32). The prognosis for fertility is very poor in women with tubal and intrauterine adhesions due to GTB, as the fallopian tubes are often blocked and scarring of the endometrial cavity affects the results of in vitro fertilization and embryo transfer (22, 27, 32, 34). Conventional methods (smear and culture) for GTB diagnosis are considered as the gold standard, but the efficacy for histopathology is low, especially in paucibacillary endometrial MTB (25, 32, 34). Further, GTB is a paucibacillary form of the disease in which smears and cultures are usually negative leading to under diagnosis of this clinical problem (23, 25).

PCR as a rapid, sensitive and specific molecular method is performed for detecting mycobacterial DNA in endometrial samples from suspected TB patients (24, 25, 39). PCR assays have abbreviated the turnaround time for definitive bacteriological detection in the laboratory to 1–2 days, besides being more sensitive than conventional methods. Based on current knowledge that laparoscopy is a valuable tool for diagnosing tubal infertility as well as GTB and the fact that PCR is very sensitive and specific f0or detecting mycobacterial infection (25, 39, 40), the present study utilized PCR technique to investigate the prevalence of GTB in a sample of Iranian women with unexplained infertility. In the present study, diagnosis of GTB in suspicious cases was made by the diagnostic laparoscopy and HSG findings. In all negative patients on laparoscopy and HSG, amplification of DNA MTB in endometrial specimen by means of PCR technique was also done to avoid missing cases.

In our study, diagnostic HSG and laparoscopy were used to screen the clinically GTB suspected subjects with abnormal tubal or uterine manifestations, and for endometrial tissue aspiration. There was no patient with positive PCR in the present study; although in another study there were 8 PCR positive patients, among 35 endoscopically normal, while 91 patients with endoscopic features suggestive of GTB did not have positive TB PCR (41).

In summary, this study has evaluated 144 unexplained infertile women with no demonstrable evidence of tubal or endometrial involvement, and it was revealed no cases with positive MTB infection in endometrial biopsy by PCR technique. Therefore, GTB could not be considered as a major factor in Iranian women with unexplained infertility.
